# Nanocomposite Design for Energy-Related Applications

**DOI:** 10.3390/nano15171334

**Published:** 2025-08-29

**Authors:** Qiu Jiang, Hanfeng Liang, Yizhou Zhang, Gang Huang

**Affiliations:** 1 Yangtze Delta Region Institute (Huzhou), University of Electronic Science and Technology of China, Huzhou 313000, China; 2 School of Materials and Energy, University of Electronic Science and Technology of China, Chengdu 611731, China; 3 State Key Laboratory of Physical Chemistry of Solid Surfaces, Tan Kah Kee Innovation, Laboratory (IKKEM), College of Chemistry and Chemical Engineering, Xiamen University, Xiamen 361005, China; hfliang@xmu.edu.cn; 4 Institute of Advanced Materials and Flexible Electronics (IAMFE), School of Chemistry and Materials Science, Nanjing University of Information Science and Technology, Nanjing 210044, China; yizhou.zhang@nuist.edu.cn; 5 State Key Laboratory of Rare Earth Resource Utilization, Changchun Institute of Applied Chemistry, Chinese Academy of Sciences, Changchun 130022, China; ghuang@ciac.ac.cn

**Keywords:** nanocomposites, energy, lithium-ion battery, thermochromic, SOFC, binder

## Abstract

Nanocomposites, which combine various nanomaterials, offer immense potential in the design of advanced materials for energy-related applications. These materials, engineered at the nanoscale, exhibit enhanced properties compared to their bulk counterparts, such as improved electrical conductivity, mechanical strength, and thermal stability. Nanocomposites have emerged as promising candidates for use in energy storage systems, including batteries and supercapacitors, by improving energy density, cycle life, and charge–discharge rates. In renewable energy technologies such as fuel cells, nanocomposites play a crucial role in enhancing efficiency and stability, which are vital for reducing costs and promoting the adoption of clean energy solutions. The unique properties of nanocomposites, such as high surface area and tunable composition, allow for the integration of multiple functionalities, making them ideal for multifunctional catalysts in energy conversion and environmental remediation. Additionally, nanocomposites enable the development of energy harvesting systems with improved performance and durability. These materials can be tailored by adjusting the composition of the nanomaterials, opening new opportunities for energy applications. The increasing research into nanocomposites continues to drive innovation in energy-related technologies, positioning them as a key enabler for sustainable energy solutions and future advancements in renewable energy systems.

Nanocomposites, materials that combine multiple nanomaterial components, have emerged as a significant area of research due to their unique properties and potential applications across a wide range of industries. When engineered at the nanoscale, materials often exhibit distinct electronic, physical, and mechanical characteristics that are drastically different from their bulk counterparts. These unique properties are particularly advantageous in the design of advanced technologies in energy systems, such as batteries, supercapacitors, solar cells, fuel cells, and multifunctional catalysts. Nanocomposites take advantage of the synergistic combination of the properties of different nanomaterials, allowing for the creation of materials with enhanced or even entirely new functionalities that cannot be achieved through the use of individual components alone. This synergy offers nanocomposites greater flexibility in terms of chemical composition, morphology, and other properties, making them suitable for highly specialized applications that require optimized performance. For example, in energy storage systems such as batteries and supercapacitors, nanocomposites provide enhanced energy density, longer cycle life, and faster charge–discharge capabilities compared to conventional materials. They offer improved efficiency and stability in solar cells and fuel cells, crucial for reducing costs and increasing the feasibility of renewable energy technologies. Additionally, nanocomposites play an essential role in multifunctional catalysts, where their unique surface properties can accelerate chemical reactions, increasing their utility in sustainable energy production and environmental remediation. The flexibility of nanocomposites allows researchers to tailor their properties by adjusting the composition of the components, opening new avenues for energy harvesting, conversion, and storage technologies. Their potential to improve existing materials and enable the development of new energy systems is transformative, positioning nanocomposites as a cornerstone for future advances in renewable energy and sustainability. As research on nanocomposites continues to evolve, their applications in novel energy-related fields are expected to expand, driving innovation in the quest for more efficient and sustainable solutions to global energy challenges. This Special Issue features a selection of six original research articles and two in-depth reviews, focusing on the latest developments, ongoing challenges, and future prospects in this evolving field.

One often-overlooked aspect of the energy storage field is its environmental impact. A prime example is the widespread use of polyvinylidene fluoride (PVDF) as a binder. These substances are notorious for their persistent and bioaccumulating pollution, particularly during production processes, leading to significant environmental concerns. To address this, Nirschl et al. investigated the potential of using silica as a substitute for PVDF as a binder for carbon black [1]. They synthesized colloidal silica particles via the Stoeber process and coated them onto carbon black using a spray flame method. The study revealed that larger silica particles, due to their higher mass, tended to deflect upon contact with carbon black aggregates, while smaller particles, especially those with a primary particle size of 10 nm, adhered more readily and sintered to the carbon black. The findings suggest that the optimal conditions for the hetero-aggregation of carbon black and silica occur when the mass ratios of silica to carbon black are equal to or below one and when the silica particles are small in size.

Regarding materials engineering for energy storage, Wang and colleagues introduced a novel three-dimensional porous nanosheet antimony/carbon (3DPNS-Sb/C) anode [2]. This composite demonstrates a reversible capacity of 511.5 mAh g^−1^ when cycled at 0.5 A g^−1^ over 100 cycles, and maintains 289.5 mAh g^−1^ at an elevated rate of 10 A g^−1^. The synthesis protocol entails a controlled hydrothermal reaction followed by thermal annealing, during which Sb nanoparticles are homogeneously generated in situ within a self-supporting organic carbon matrix. The uniform dispersion of Sb is ascribed to the facile reduction of sodium antimonate and the strong interfacial affinity afforded by the carbon network. Consequently, the resulting 3DPNS-Sb/C composite leverages both the structural advantages of its polymer-derived framework and the enhanced electrical conductivity imparted by the carbon scaffold. Similarly, Ma et al. synthesized a series of Sn-doped Li_1.3_Al_0.3_Sn_x_Ti_1.7−x_(PO_4_)_3_ (LATP-xSn) ceramic electrolytes via a conventional solid-state route, systematically varying the Sn^4+^ content to probe its effects on crystal structure and ion-transport characteristics [3]. To mitigate interfacial resistance between the ceramic and polymer phases, they fabricated a sandwich-type composite electrolyte by casting a PEO-based polymer precursor onto both faces of the modified LATP pellet, rather than blending the two constituents homogeneously. This bilayer architecture was shown to markedly lower solid–solid interface impedance, thereby enhancing overall electrochemical performance.

Thermochromic materials—which reversibly alter their optical or electrical properties in response to temperature changes—hold significant promise for energy-efficient technologies, adaptive optics, and environmental sensing. By dynamically modulating solar transmittance or reflectance, thermochromic coatings can reduce building energy consumption for heating and cooling without relying on external power, thereby lowering greenhouse gas emissions and operational costs. In its monoclinic form, vanadium dioxide (VO_2_) undergoes a reversible phase transition to the rutile structure upon heating, a transformation accompanied by a sharp drop in near-infrared transmittance that makes it an attractive candidate for smart window coatings. Zhao et.al reported a straightforward one-step annealing procedure to synthesize VO_2_ powders [4]. Following synthesis, the particles were rapidly quenched in either deionized water or ethanol to introduce controlled surface lattice distortions. The resulting composite films exhibited pronounced localized surface plasmon resonance (LSPR) effects, leading to a significant enhancement in solar-energy modulation performance compared to undistorted VO_2_ coatings.

Molecular oxygen, upon activation by visible light, generates radicals with substantial oxidation capabilities, offering significant potential for environmental remediation. In a noteworthy study, Zhang et al. demonstrated that coupling two-dimensional (2D) CdIn_2_S_4_ with 2D graphitic carbon nitride (g-C_3_N_4_) represents an efficient strategy to enhance the photocatalytic activity of CdIn_2_S_4_ while simultaneously reducing the reliance on cadmium species [5]. Their research reported the successful construction of 2D/2D CdIn_2_S_4_/g-C_3_N_4_ nanocomposites with varying weight ratios, achieved through a straightforward and cost-effective electrostatic self-assembly technique. This approach not only improves the photocatalytic performance but also offers a sustainable method for reducing the environmental impact associated with Cd-based materials.

Transition metal nitrides have emerged as materials of considerable research interest due to their hybrid bonding character—combining metallic, ionic, and covalent interactions—which underpins their remarkable mechanical and electronic performance. In a recent first-principles investigation, Zhang and co-workers explored the impact of manganese substitution on the cubic phase of molybdenum nitride (d-MoN:Mn) [6]. Through detailed electronic structure calculations and comparative analysis of multiple spin arrangements, they unraveled the mechanisms through which Mn dopants induce magnetic behavior and mediate exchange interactions. Their results reveal how the introduction of Mn modifies the host’s band structure and fosters long-range magnetic coupling among dopant sites.

In addition to the recent experimental advancements in the field, two comprehensive reviews have made substantial contributions by providing in-depth analyses of specific materials relevant to energy applications. One of these reviews focuses on the two prominent families of oxygen-conducting electrolytes: doped lanthanum aluminates (LaAlO_3_) and lanthanum gallates (LaGaO_3_) [7]. These materials are of particular interest due to their crucial role in solid oxide fuel cells (SOFCs), where high ionic conductivity and chemical stability at elevated temperatures are essential. The review meticulously examines the preparation methods of these electrolytes, their chemical stability, thermal behavior, and transport properties, all of which are deeply intertwined with the performance characteristics required for efficient SOFC operation. The second review delves into the promising field of inks based on two-dimensional (2D) MXene sheets, which have garnered significant attention due to their unique properties and versatile applications [8]. This review comprehensively explores the recent developments in the synthesis of MXene-based inks, highlighting their potential for use in various applications such as flexible electronics, sensors, energy storage devices, and as conductive materials in printed electronics. The properties, advantages, and limitations of MXene inks are thoroughly discussed, with particular attention to their future potential in the rapidly evolving field of additive manufacturing and printed electronics.
